# Supplementary material for: A Single Bout of Ultra-Endurance Exercise Reveals Early Signs of Muscle Aging in Master Athletes
Source: Int J Mol Sci. 2022 Mar 28;23(7):3713. doi: 10.3390/ijms23073713 (PMC8998696; doi:10.3390/ijms23073713)
Supplement: Supplementary file 1 [file Table S3.pdf › Table S3.pdf]

Table S3. Functional class scoring (FCS) of pathways affected by 24TR in 50Y runners.

Name	Type	*P*	n
Glycolysis Gluconeogenesis	C	0.024	15
Integrin	C	0.040	5
Oxidative phosphorylation	K	0.043	46
Respiratory electron transport	R	0.017	39

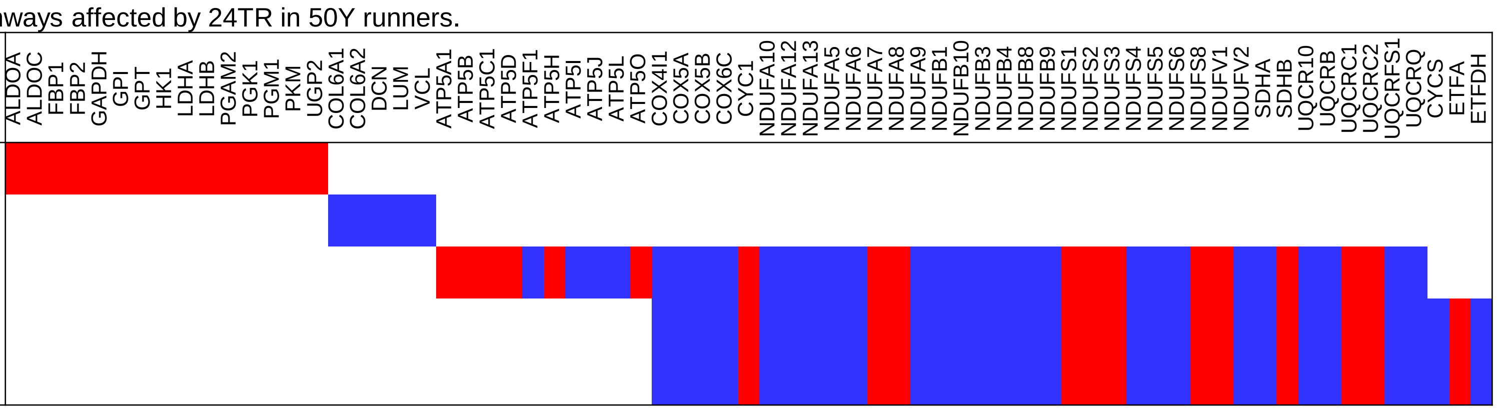

C, ConsensusPathDB; K, KEGG Pathway; R, Reactome Pathway; *P*, adjusted *P* value; n, number of proteins identified in the pathway.Red and blue indicate higher and lower mean protein levels after 24TR.
