# Supplementary material for: A Single Bout of Ultra-Endurance Exercise Reveals Early Signs of Muscle Aging in Master Athletes
Source: Int J Mol Sci. 2022 Mar 28;23(7):3713. doi: 10.3390/ijms23073713 (PMC8998696; doi:10.3390/ijms23073713)
Supplement: Supplementary file 1 [file ijms-23-03713-s001.zip › Figure S1.pdf]

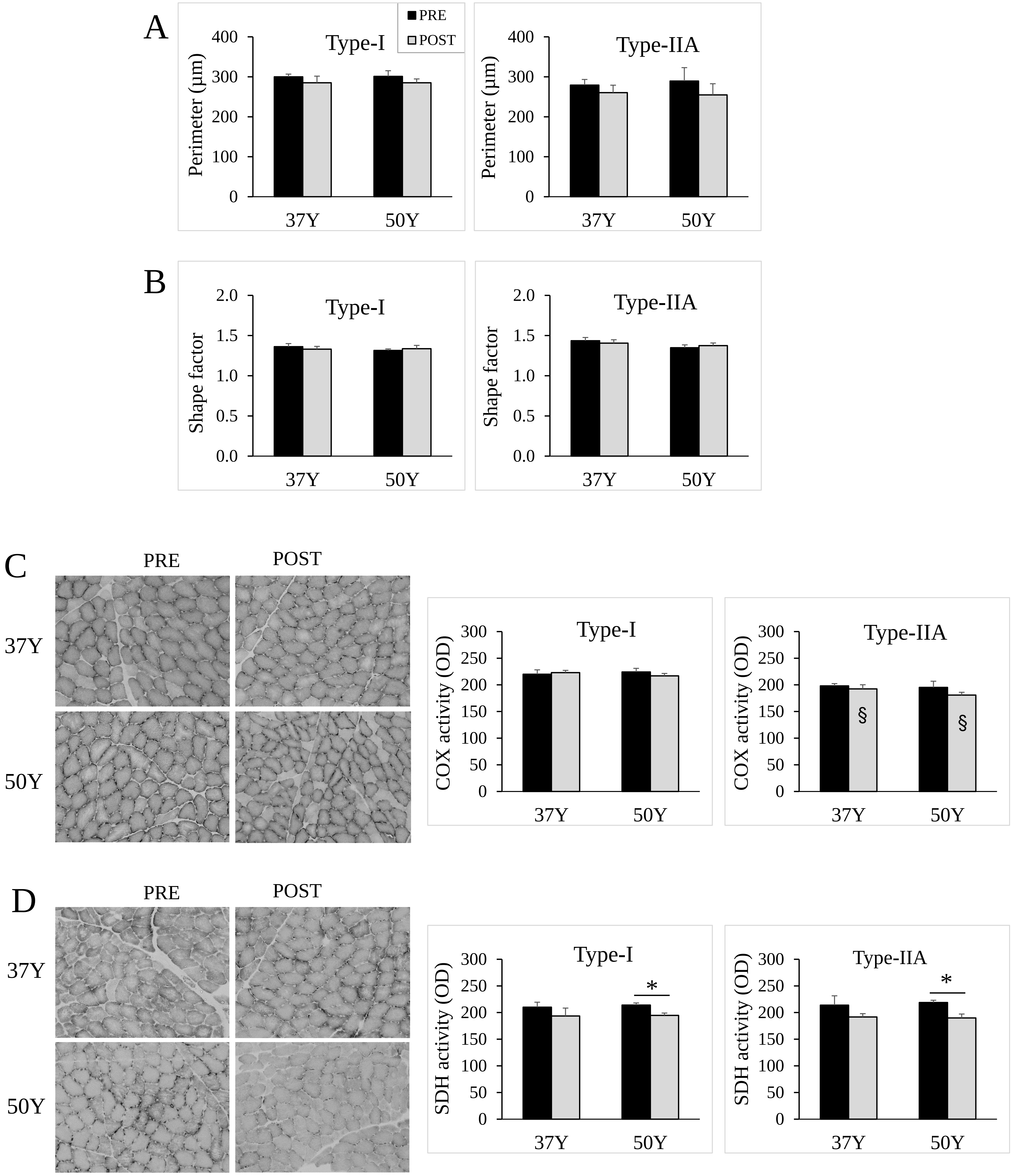

Figure S1. Fiber morphometry and mitochondrial histoenzymology. (A) Mean perimeter; (B) mean shape-factor; (C) representative images of stained sections and normalized cytochrome c (COX) activity; and (D) representative images of stained sections and normalized succinate deshydrogenase (SDH) activity, of type-I (left) and type-IIA (right) fibers in 37Y and 50Y runners PRE (black bars) and POST (grey bars) 24TR. Results are means $\pm$ *SE* (*n*=4-6). § $P < 0.05$ between type-I and type-IIA fibers; * $P < 0.05$ between PRE and POST.
