# Supplementary material for: A Single Bout of Ultra-Endurance Exercise Reveals Early Signs of Muscle Aging in Master Athletes
Source: Int J Mol Sci. 2022 Mar 28;23(7):3713. doi: 10.3390/ijms23073713 (PMC8998696; doi:10.3390/ijms23073713)
Supplement: Supplementary file 1 [file ijms-23-03713-s001.zip › Supplementary Methods.pdf]

Supplementary Methods: Proteomics

1. Muscle Homogenate Preparation

Ten serial cross-sections (30 µm thick) were homogenized at 4 °C in a solubilisation buffer containing 8.3 M urea, 2 M thiourea, 2% CHAPS, 1% dithiothreitol and protease inhibitor cocktail, and centrifuged for 10 min at 10,000 g. The supernatant was mixed with 0.33 volume Laemmli loading buffer 4X (Amresco, VWR International, Fontenay-sous-bois, France) and heated at 95 °C for 5 min. SDS-PAGE (12% acrylamide) was performed using a Mini-Protean IV electrophoresis unit (BioRad). Samples were loaded at 70 µg protein per lane. To concentrate the samples, gels were run at 80 V until the dye front reached the bottom of the concentration gel. Gels were fixed for 10 min with 30% ethanol, 5% acetic acid, stained for 20 min in Coomassie brilliant blue R-250, and destained for 10 min in fixation solution and then in distilled water. Excised lanes were reduced with 10 mM dithiotreitol, alkylated with 55 mM iodoacetamide (both in 50 mM ammonium bicarbonate), and incubated in 25 mM ammonium bicarbonate with acetonitrile (v/v) until destaining. After incubation in 100% acetonitrile, gel pieces were dried in a vacuum SpeedVac. They were further rehydrated with 30 µl of a trypsin solution (10 ng/µl in 25 mM ammonium bicarbonate), and finally incubated overnight at 37 °C. Peptide extraction was optimized by adding 24 µl of 100% acetonitrile followed by 10 min of sonication. The trypsin digests were dried in a vacuum SpeedVac and stored at -20 °C in a solution of 2% acetonitrile, 0.05% trifluoroacetic acid, before nano-liquid chromatography coupled to tandem mass spectrometry (nano-LC-MS/MS).

2. Nano-LC-MS/MS Analysis

The peptides mixtures were analysed using an Ultimate 3000 system (Dionex, Voisins le Bretonneux, France) coupled to an Impact II (Bruker, Champs sur Marne, France) with a CaptiveSpray ion source (Bruker). Eight microliters of each hydrolysate were loaded on a C18 pre-column (300-µm inner diameter × 5 mm; Dionex) at 30 µl/min in 97.9 % H_2O, 2% acetonitrile, and 0.1% trifluoroacetic acid. After 6 min of desalting, the pre-column was switched on line with the analytical C18 column (75 µm inner diameter × 50 cm, 2.6µm; Accucore, nanoViper C18) equilibrated in 96% solvent A (99.9% H_2O, 0.1% formic acid) and 4% solvent B (99.9% acetonitrile, 0.1% formic acid). Peptides were eluted using a 4 to 30% gradient of solvent B during 180 min at a 250 nl/min flow rate. The eluate was electrosprayed into the mass spectrometer through a CaptiveSpray ion source. The QTOF impact II was used in a CID mode to acquire a maximum number of MS/MS possible in three seconds after the full MS scans. At the end of each LC-MS/MS analysis, the raw files (.d) were converted into .mgf format and then imported into ProteinScape (Bruker) and Progenesis LC-MS software (version 4.1, Nonlinear Dynamics, Newcastle, UK).

3. Protein Identification

Mascot was used for database search (http://www.matrixscience.com). For protein identification, the Uniprot Taxonomy Human protein database was used. The following parameters were considered for the searches: peptide mass tolerance was set to 500 ppm,

fragment mass tolerance was set to 0.5 Da and a maximum of two missed cleavages was allowed. Variable modifications were methionine oxidation (M) and carbamidomethylation (C) of cysteine. Protein identification was considered valid if at least one peptide with a statistically significant Mascot score assigned it (with Mascot score ≥ 36 for p-value < 0.05 with a False Discovery Rate (FDR) at 1%). Identification of proteins based on one peptide was accepted after checking the correct assignment of fragment ion matches (at least three consecutive fragments b/y, match peaks well above the background noise). Identifications not satisfying these defined criteria were rejected.

4. Label-free Protein Quantitation

The profile data of the MS scans and MS/MS spectra were converted to peak lists comprising m/z and abundance with Progenesis QI using a proprietary algorithm. One sample was automatically set as the reference, and the retention times of all other samples were automatically aligned to create maximal overlay of the two-dimensional feature maps. Features with only one charge, with retention time windows lower than 6 s or with retention time <20 min or >80 min were excluded from further analyses. Using all remaining features, a normalization factor was calculated for each sample, to correct experimental variation. All unique validated peptides (with Mascot score ≥ 36 for p-value < 0.05) of an identified protein were included for quantification, and the total cumulative abundance was calculated by summing the abundances of all peptides allocated to the respective protein. Analysis was performed using the normalized abundances across all runs. The mass spectrometry proteomics data have been deposited to the ProteomeXchange Consortium via the PRIDE partner repository with the dataset identifier PXD006840.
