# Supplementary material for: A Single Bout of Ultra-Endurance Exercise Reveals Early Signs of Muscle Aging in Master Athletes
Source: Int J Mol Sci. 2022 Mar 28;23(7):3713. doi: 10.3390/ijms23073713 (PMC8998696; doi:10.3390/ijms23073713)
Supplement: Supplementary file 1 [file ijms-23-03713-s001.zip › Table S1.pdf]

Table S1. Effect of 24TR on muscle fiber microvascularisation

		37Y		50Y	
		PRE	POST	PRE	POST
CD (mm^{-2})		367 ± 27	338 ± 12	389 ± 22	403 ± 25
CAF	I	5.03 ± 0.27	5.00 ± 0.27	5.74 ± 0.33	5.37 ± 0.30
	IIA	4.14 ± 0.41	4.04 ± 0.32	4.21 ± 0.52 §	3.59 ± 0.49 §
C/Fi	I	2.03 ± 0.15	2.02 ± 0.13	2.35 ± 0.14	2.17 ± 0.14
	IIA	1.62 ± 0.19 §	1.65 ± 0.14	1.64 ± 0.24 §	1.38 ± 0.19 §
CFPE (nm^{-1})	I	7.21 ± 0.46	6.94 ± 0.19	8.05 ± 0.55	7.95 ± 0.49
	IIA	6.02 ± 0.64 §	6.05 ± 0.34	6.14 ± 0.54 §	5.72 ± 0.52 §

CD, capillary density; CAF, fiber-type specific capillary-to-fiber ratio; C/Fi, individual capillary-to-fiber ratio; CFPE, capillary-to-fiber perimeter. Data are means ± SE for groups 37Y and 50Y, either PRE or POST 24TR. $^{\S}P < 0.05$ vs. type-I fiber.
