# Supplementary material for: A Single Bout of Ultra-Endurance Exercise Reveals Early Signs of Muscle Aging in Master Athletes
Source: Int J Mol Sci. 2022 Mar 28;23(7):3713. doi: 10.3390/ijms23073713 (PMC8998696; doi:10.3390/ijms23073713)
Supplement: Supplementary file 1 [file ijms-23-03713-s001.zip › Table S2.pdf]

Table S2. Label-free quantitative protein profiling of *vastus lateralis* proteomes

Accession	Symbol	Protein	Peptides	Unique peptides	Confidence score	Fold change	Ttest	F
37Y versus 50Y PRE-24TR								
P40925-3	MDH1	Isoform 3 of Malate dehydrogenase, cytoplasmic	20	12	1307	-1.8	0.001	0.15
B4DXY7	ALDH9A1	4-trimethylaminobutyraldehyde dehydrogenase	1	1	63	-2.6	0.002	0.34
F8W7Q4	FAM162A	Protein FAM162A	1	1	34	-3.8	0.004	0.58
Q96DC0	DCI	DCI protein	2	2	114	-1.7	0.005	0.80
PRE versus POST-24TR for 37Y runners								
Q6P1W5	C1orf94	Uncharacterized protein C1orf94	1	1	29	1.9	0.002	0.34
P10644	PRKAR1A	cAMP-dep. protein kinase type I-α regulatory	6	3	218	-2.4	0.004	0.69

F, number of expected false positive
